# The social grounds of self-tracking in insurance: A mixed-method approach to adoption and use

**DOI:** 10.1177/20552076231180731

**Published:** 2023-06-07

**Authors:** Bastien Presset, Fabien Ohl

**Affiliations:** 1 Institute of Sport Sciences, Faculty of Social and Political Science, University of Lausanne, Lausanne, Switzerland; 2 Department of Technology and Society Studies, Faculty of Arts and Social Sciences, Maastricht University, Maastricht, The Netherlands

**Keywords:** mHealth, sociology, public health, disease, lifestyle, Bourdieu, health inequalities, self-tracking, insurance, exercise, digital divide

## Abstract

Scholars have explored the role of self-tracking in mediating people's values, perceptions, and practices. But little is known about its institutionalised forms, although it is becoming a routine component of health policies and insurance programs. Furthermore, the role of structural elements such as sociodemographic variables, socialisations, and trajectories has been neglected. Using both quantitative (*n*  =  818) and qualitative (*n*  =  44) data gathered from users and non-users of an insurance program's self-tracking intervention, and drawing from Bourdieu's theoretical framework, we highlight the impact of users’ social background on the adoption and use of the technology. We show that older, poorer, and less educated individual are less likely to adopt the technology, and describe four prototypical categories of users, *the meritocrats*, *the litigants*, *the scrutinisers* and the *good-intentioned*. Each category displays different reasons and ways to use the technology that are grounded in users’ socialisations and life trajectories. Results suggest that too much emphasis may have been put on self-tracking's transformative powers and not enough on its reproductive inertia, with important consequences for both scholars, designers, and public health stakeholders.

## Introduction

Insurance companies and public health institutions are gradually investing in digital self-tracking^a^ (ST) interventions.^1^ Their engagement is based on stereotyped and hyperbolic promises;^b^ notably that ST will reduce the burden of noncommunicable diseases by modifying users’ health behaviours.^2,3^ Such claims are at the centre of vivid and polarised debates, although empirical accounts of ST in institutional contexts remain scarce.^4^

Unlike personal, idiosyncratic forms of tracking, institutionalised and financially rewarded ST – for example, as part of an insurance program – involves its valorisation by a major health institution and its integration in a health-economy and the institutional governance of health behaviours. Behaviour-based programs and tracking technologies that deliver financial bonuses and premium reductions are increasingly being offered to large populations of policyholders, thereby transforming their relations with insurance companies.^5^ This raises critical – and so far understudied – issues, notably regarding the impact of those digital programs on health inequalities.^6,7^

We aim to address this by using data gathered from actual users of a digital ST program developed by an insurance company. The ST application is called myStep and was developed by one of Switzerland's largest insurance company.^c^ It offers a premium reduction to users who reach a daily step objective.^d^ In this paper, we enquire the patterns of adoption and use of this technology, and, by extension, question ST's entanglement with health inequalities. We first question whether the adoption of the intervention is homogenous by analysing sociodemographic differences between users and non-users. We then explore differences as to how groups of users appropriate the technology in daily life. Thereby, we tackle the two identified loci of digital inequalities: adoption and use.^8^

Our results indicate that adoption is not homogenous and may lead to patterned inequalities. Regarding use, we describe four prototypical categories of appropriation, *the meritocrats*, *the litigants*, *the scrutinisers* and the *good-intentioned*. In both cases, adoption, and use, we demonstrate the central role played by social conditions, socialisations, and trajectories. We then discuss the importance of those results for public health institutions, insurances, and scholarship, stressing the fact that ST, although framed as mundane and inclusive by its proponents, bears the risk of transmuting social inequalities into a personal responsibility of being a ‘responsible citizen’.

## Literature review: pasts as blindsight of ST studies

ST – defined as ‘practices in which people knowingly and purposively collect information about themselves, which they review and consider applying to the conduct of their lives’ – has received much attention in both scholarly literature and news media.^9^^(p2)^ It is understood to be both the product and productive of ‘broader social, cultural and political processes’^9^^(p1)^ that emphasise surveillance,^10,11^ datafication,^12^ healthism, liquid or late modernity^13,14^ or neoliberalism.^15^

ST is often understood as a practice that mediates surveillance and discipline while giving opportunities for resistance and creativity. This tension can be traced back to the Foucauldian dialectic – central in ST literature – of governmentality and critique.^16^ Most empirical accounts have followed this path, showing how ST is both an opportunity to change oneself and a disciplining constraint; a possibility to create and resist in the midst of a field of power.^17–19^ Scholars have, thus, decisively complicated the Manichean notion of ST to be either constricting or liberating, thereby transforming a polarised opposition often found in discourses into a dynamic duality.

By both imposing cultural norms and enhancing reflexive changes, ST practices are central to the production of selves.^9^ Science and technology studies and post-phenomenology theories have been used to explore the mechanics of the process by which user perceptions, affects, values and practices are mediated.^20^ Selves are reconfigured as products of dynamic interactions within sociomaterial networks^21,22^ and scholars have relied on concepts such as ‘data double’^23^ or ‘laboratory of the self’^19^ to fruitfully analyse such processes. Emphasis has also been put on the active work of users who perform ST – and make sense of it – in their own ways (Ajana 2017, Pols et al. 2019; Spotswood et al., 2020).^24–26^ Scholars have reinscribed ST within its material, affectual and phenomenological networks, showing how such networks are transformed and, thus, elaborating some of ST's transformative effects.^27^

Emphasis has thus been put on the mediation of digital selves and the transformative effects of the technology. But – as confirmed by a recent review – little attention has been paid to users’ pasts and to the broader social grounds of ST.^28^ ST users are typically presented in an ahistorical social vacuum, as if their dispositions and positions in social space did not affect their uses. The prevalence of the aforementioned theoretical frameworks may have played a part in obscuring the role of social factors, something that we come back to in the discussion. Two studies are exceptions to this rule. A quantitative study in Germany found that trackers were generally younger, had ‘relatively high school and job qualifications’ and had a ‘rather high proportions of both psychopathologies and/or somatic pathologies’.^29^^(p9)^ A study based on interviews with French trackers suggests that ‘individuals from affluent or intermediate social milieus’ are most likely to use tracking apps and that tracking may be enabled by ‘specific position in life course’ and constrained – for people from ‘poorer milieus’ – by a feeling of ‘cultural illegitimacy’.^30^^(p5)^

These studies tackled the social grounds of self-tracking, which remain largely understudied. This neglection has the consequences of concealing inequalities, a topic that is even more pressing when ST becomes institutionalised, in our case in the context of social insurance. The rare studies that tackle the social determinants of ST include different tracking technologies that are used in different non-institutionalised contexts. By analysing patterned differences in the adoption and use of a ST technology developed by an insurance company, by using a mix of quantitative and qualitative data, and by drawing from Bourdieu's conceptualisation of the social reproduction of inequality, we aim to contribute to and stimulate that crucial emerging field of research.

## Theoretical framework: Bourdieu and dispositions

**Figure 1.**
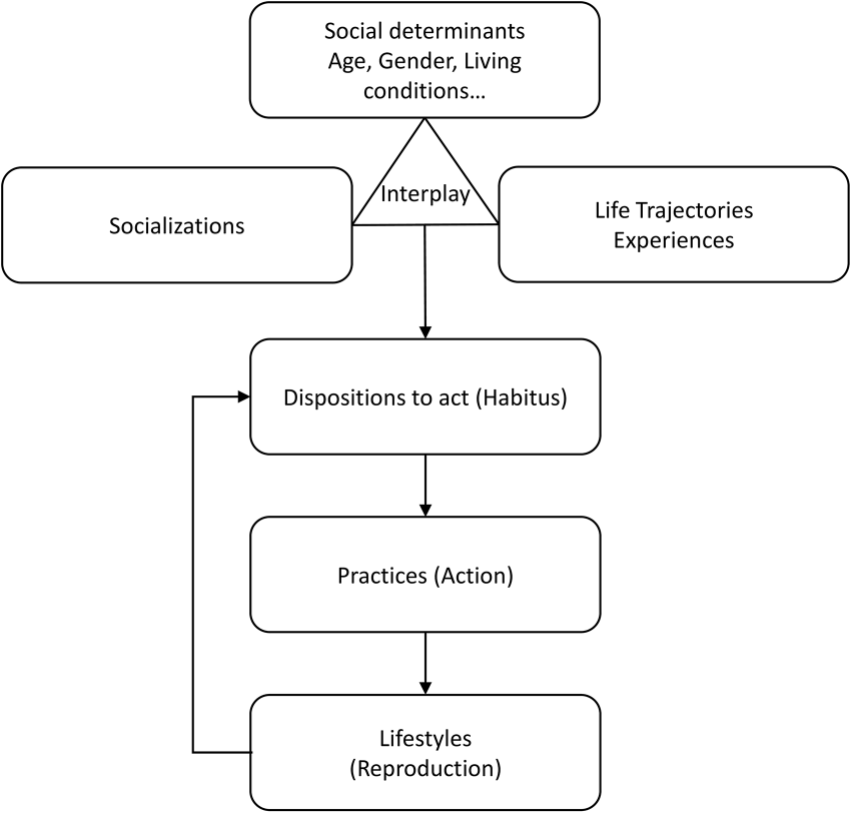
Schematic representation of our dispositionnalist framework. Adapted from Bourdieu^44^^(p171)^ and Cockerham.^35^

Our research is based on the following empirical observation: users’ social background influences their adoption and use of ST technologies.^29–32^ Drawing from Bourdieu, we aim to conceptualise this background as a collective and continuous process of incorporation of dispositions.^33^ Dispositions are embodied culture, ‘structured structures predisposed to operate as structuring structures, that is, as principles which generate and organize practices and representations’.^34^^(p53)^ They are the product of a dynamic interplay between structural variables (age, gender, income, education, etc.), socialisations and life trajectories. This dynamic interplay leads to the incorporation of dispositions to act that are exteriorised in practices (such as ST) and lifestyles.^35^Figure 1 heuristically summarises this process.

Bourdieu's framework has been criticised as deterministic. But this is forgetting that the system of dispositions is open, it ‘is constantly subjected to experiences, and therefore constantly affected by them in a way that reinforces or modifies its structures’.^36^^(p133)^ Dispositions may both evolve and be plural (or even contradictory) and are activated in relation to contexts.^37^ Moreover, dispositions should be understood in a probabilistic way, they enable or constrain more than force or forbid. It is in that probabilistic sense that we conceptualise dispositions’ relation to inequalities. If they tend to further the likelihood of a harmful practice or pattern to reproduce itself, dispositions reproduce and foster inequalities. This process is consistently identified with regards to most health outcomes and practices.^38^ It is also the conceptual root of digital inequalities studies.

Digital inequalities are understood as ‘a hierarchy of access to various forms of technology in various contexts, resulting in differing levels of engagement and consequences’.^8^^(p351)^ This definition is generally broken down into different tenets. First, not everyone has access to, or is disposed (not everyone is equally likely) to adopt a particular technology. In the case of mobile health, this is evidenced by quantitative studies that have shown how younger people with a higher education and a higher income are statistically more likely to use mobile health applications.^29,39,40^ Second, not everyone uses a technology in the same way, and certain uses can be more beneficial than others.^41^ In other terms, authors distinguish between two moments in the life of a technology: adoption (acquiring the technology) and use (using the acquired technology), and these moments highlight the usual loci of inequalities.^8^ In both cases, sociodemographic variables are identified as key factors. Using Bourdieu's framework to explore these divides allows us to elaborate the role of people's social backgrounds in the production and reproduction of harmful differences (inequalities) in the adoption and use of a ST technology in insurance.

## Methods

This research lies in the field of critical social sciences and is based on empirical data. It follows a mixed-methods perspective and combines questionnaire and semi-directive interview data to analyse inequalities in insurance ST. The samples, measures, and analyses for both questionnaire and interviews are presented hereunder.

### Study sample

Questionnaires were sent by email to 20,000 Swiss supplementary insurance customers who were eligible to use myStep. As this was sociological research on adult subjects that does not entail sensitive topics (non-medical survey with a focus on physical activities), it did not fall within the scope of submission to the local ethics committee. The email was sent to all French speaking myStep users. For non-users, we divided the population according to gender and three age groups (18–39; 40–64; >65) and sent the email to a minimum of 700 customers for each of the 6 categories. A total of 231 users of the myStep application and 840 non-users completed the questionnaire (response rate: 5.3%). Forty-one users and 212 non-users were excluded because they did not fully complete it. Table 1 summarises the sociodemographic information of the 818 participants included in the study. The questionnaire was divided in five parts (physical activities – technologies – myStep – tracking in insurance and demographics). However, only the demographic information is used in this article.

**Table 1.** Summary of the questionnaire sample's sociodemographics.

	Full sample (*n* = 818)		myStep users (*n* = 190)		Non-users (*n* = 628)	
Women (*n*, %)	443	54.16	108	56.84	335	53.34
Age (*M*, SD)	44.91	15.48	41.01	12.97	46.09	15.90
Income (*n*, %)						
Very low	84	10.27	9	4.74	75	11.94
Low	166	20.29	43	22.63	123	19.59
Middle	163	19.93	35	18.42	128	20.38
High	168	20.54	45	23.68	123	19.59
Very high	237	28.97	58	30.53	179	28.50
Education (*n*, %)						
Low	18	2.20	4	2.11	14	2.23
Middle	259	31.66	66	34.74	193	30.73
High	72	8.80	18	9.47	54	8.60
Very high	469	57.33	102	53.68	367	58.44
Time of use since adoption in months (*M*, SD)			14.96	12.22		

Users who filled out the questionnaire were asked to participate in an interview. Those who accepted did so in writing. They were then sent an email that detailed the rationale of the study and the interview process. They were informed about the context and goal of the study and the fact that they could opt out at any moment and refuse to answer any question. They chose the location of the interviews. A total of 21 were interviewed by the first author (PhD student at the time of the study). Selection was based on diversity (gender, age, education, and income). It should also be noted that 23 interviews were previously conducted with users of a myStep prototype. We decided to include those 23 interviews in our analysis for the small differences between the prototype and myStep do not influence the results presented in this paper. Details on the prototype and the interviews with prototype users can be found in a previous paper.^31^ Questionnaires were gathered in august 2019 and interviews between September and December 2019. Table 2 summarises the sociodemographic information of the 44 interviewees.

**Table 2.** Summary of the interview sample's sociodemographics.

No.	Profession	Age	Education	Income	Gender	Months of use	Use category
1.1	Retired (insurance manager)	64	–	–	Man	–	Good-intentioned
1.2	Retired (home care director)	73	–	–	Woman	–	Good-intentioned
1.3	Crisis manager	31	–	–	Man	–	Good-intentioned
1.4	Manager	66	–	–	Woman	–	Good-intentioned
1.5	Banker	47	–	–	Man	–	Meritocrat
1.6	Informatician	54	–	–	Man	–	Good-intentioned
1.7	Engineer	38	–	–	Man	–	Scrutiniser
1.8	Engineer	47	–	–	Man	–	Scrutiniser
1.9	Senior manager	56	–	–	Man	–	Unclassifiable
1.10	Banker	70	–	–	Man	–	Meritocrat
1.11	Retired (consulting)	68	–	–	Man	–	Unclassifiable
1.12	Nurse	41	–	–	Woman	–	Litigant
1.13	Informatician	55	–	–	Man	–	Scrutiniser
1.14	Salesman (ex-engineer)	62	–	–	Man	–	Scrutiniser
1.15	Office worker	27	–	–	Woman	–	Good-intentioned
1.16	Graphist	48	–	–	Woman	–	Good-intentioned
1.17	Banker	53	–	–	Man	–	Meritocrat
1.18	Communication	42	–	–	Woman	–	Litigant
1.19	Cook	46	–	–	Man	–	Good-intentioned
1.20	Manager	58	–	–	Woman	–	Meritocrat
1.21	Business consultant	29	–	–	Woman	–	Meritocrat
1.22	Medical secretary	57	–	–	Woman	–	Good-intentioned
1.23	Office worker	28	–	–	Woman	–	Good-intentioned
2.1	Sales manager	51	Very high	Very high	Man	5	Meritocrat
2.2	Banker	46	Very high	Very high	Man	18	Meritocrat
2.3	Finance Specialist	45	Very high	Very high	Woman	20	Meritocrat
2.4	Consulting	54	Very high	Very high	Woman	4	Good-intentioned
2.5	Laboratory technician	70	Middle	High	Man	2	Scrutiniser
2.6	Expert in information security	64	Very high	High	Man	32	Scrutiniser
2.7	Project manager in engineering	33	Very high	Very high	Man	12	Scrutiniser
2.8	Informatician	46	Middle	Medium	Man	18	Good-intentioned
2.9	School teacher	37	Very high	High	Man	24	Good-intentioned
2.10	Nurse	45	Middle	Low	Woman	5	Good-intentioned
2.11	Office worker	58	Middle	High	Man	2	Good-intentioned
2.12	Office worker	50	Low	–	Woman	3	Good-intentioned
2.13	Team manager	–	–	–	Man	–	Good-intentioned
2.14	Team manager	49	Middle	Very high	Man	16	Good-intentioned
2.15	Taxi driver	68	Middle	Low	Man	24	Litigant
2.16	Police officer	33	Middle	Very high	Man	6	Litigant
2.17	Retired (office worker)	75	Low	Low	Woman	1	Litigant
2.18	School teacher with direction responsibilities	31	Middle	High	Man	12	Litigant
2.19	Law school student	25	Very high	Very low	Man	16	Litigant
2.20	Office worker	37	Middle	Low	Woman	8	Good-intentioned
2.21	Sports trainer	51	Middle	–	Man	24	Litigant

### Measures

Age, gender, income, and education were measured in the questionnaire. Income categories were based on the 2018 Swiss Earnings Structure Survey by the Federal Office of Statistics (OFS): <4000 CHF (very low); between 4000 and 6000 CHF (low); between 6000 and 8000 CHF (middle), between 8000 and 10,000 CHF (high) and >10,000 CHF (very high). Education measures reflected the structure of the Swiss education system: primary and secondary compulsory education (low), upper secondary professional education (middle), upper secondary preuniversity education (high), tertiary level professional education and higher education/university (very high).

The interviews were conducted by the principal author and covered five main themes: practical software use; use of the data; history with tracking technologies and myStep; motivation; and health system. The interview guide (Table 3) offered considerable space for emerging themes and was adapted, when necessary, in accordance with grounded theory.^42^ Its content was based on a prior round of interviews with other users of a ST tool.^31^ Interviews and data analysis were done in French and German, quotations used in the article were translated by the authors. According to grounded theory principles, we stopped interviewing new subjects once data saturation was achieved, that is once additional interviews stopped providing new insights into the emerging categories.^42^

**Table 3.** Interview grid.

Category	Subcategory	Question
*Individual*	–	Who are you? Work, sport, family, education…
*Use*	Use	Can you show me your data? Can you explain it to me?
	Adoption	When did you start using the application? Why?
	Daily use	How do you use the application daily?
	Temporality	Has your use changed with time?
	Auto-evaluation	Have you learned to evaluate/measure your steps?
	Active day	What is an active day for you? Has it changed with the application?
	Less active day	What is a less active day? Has it changed with the application?
	Optimisation	Do you seek to optimise your behaviour?
	Numbers	What do you think of your numbers?
	Sharing	Do you discuss the application and step-counting with others? Who? How?
	Opinions	What do people think of your use of the application?
	Habits	Have you changed your habits? Which ones? Temporality?
*Relation to sports/physical activities*	Volume	Do you practice sports/physical activities?
	Socialisation	Did you practice sport/physical activities young? Were/are your parents active?
	Values	Why do you engage in sport/physical activities?
	Competition	Do you take part in competitions? Do you enjoy it? Why?
	Technology	Do you use technologies to measure sport? Which ones and what do you measure?
*Relation to health*	Care	Is health important to you? What do you do for your health?
	Socialisation	Has your relation to health evolved? How was it in your family?
	Health	Have you encountered health problems? And people that are close to you?
	Society	Do you think people should care more about their health?
*Relation to institutions*	Health system	What do you think of our health system and health insurance system?
		What do you think of measurement programs in insurance (such as mystep)? Risks? Benefits?
		Are individuals responsible for their own health?
*Relation to technologies*	Technologies	Do you enjoy technology? Which ones do you use daily?
	Potential	Do you think we should use more or less technologies? Why?
	Health	What do you think of technologies in health? Examples, reasons.
	Data and security	Are you confident/afraid regarding data-sharing? Who do you (not) trust?
*Relation to self*	General	Are you generally relaxed or stressed? How do you deal with work?
	Organisation	Are you generally organised? How?
	Performance	Do you think that performance is central in society? Opinion.
*Ending*		Do you have questions? Do you have something to add?

### Analysis

Statistics were calculated using SPSS software. Binary logistic regression assesses the relationship between a binary dependent variable and a set of (binary or nonbinary) independent variables.^43^ Thus, we distinguished users from non-users, using the non-user group as the reference category. Gender was entered as a categorical factor, education and income were entered as ordinal variables, and age was entered as a continuous variable. Odds ratios refer to the likelihood of using the application. Hence, numbers greater than 1 point to a greater likelihood of using myStep, whereas numbers lower than 1 point to a lower likelihood of using myStep.

**Figure 2.**
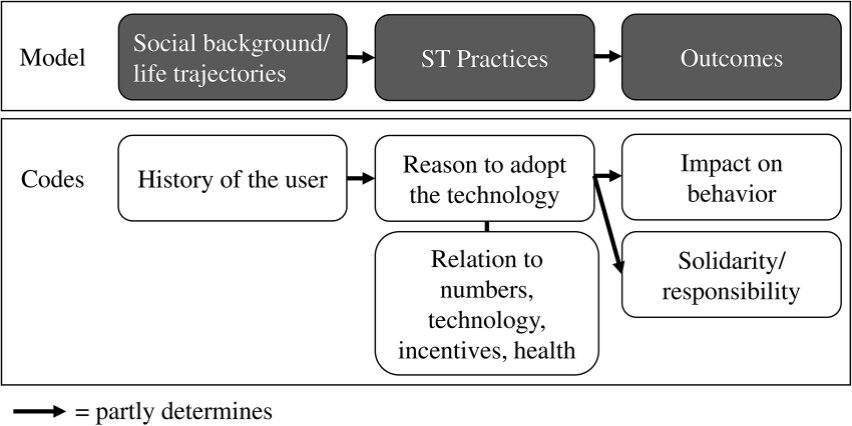
Model (top) and relations between codes (bottom).

The interview data were coded by the main author with QSR International's Nvivo 11 software, following the principles of grounded theory.^42^ An initial coding sequence (open coding), based on prior research,^31^ led to the identification of the following codes: impact on behaviour, reason to adopt the technology (which was composed of the codes: relation to physical activities, relation to numbers, relation to technology, relation to incentives, and health), solidarity/responsibility, limits of the application, and history of the user. We used Nvivo's cross comparison functions to perform axial and selective coding, that is, using the relations between codes to build concepts and a model.^42^ This led us to the following model: users’ social background (social conditions, socialisations, and life trajectories) seems to condition prototypical ways to adopt and use the technology and lead to different outcomes (see Figure 2).

## Results

### The patterned adoption of ST technology, results of the binary logistic regression

The logistic regression provides clues as to how social conditions influence the likelihood of adopting *myStep*. Table 4 summarises its results. myStep users are significantly more likely to be younger, have high education and have a high or very high income. These three factors are typically identified as significant by research on mobile health technologies^39,40^ and recent studies on ST.^29,30^ Descriptive statistics suggest that the typical user is 42  ±  12 years old, with a university degree (very high education). Regarding income, the most impoverished people (<4000 CHF/ approx. 4300 US$, December 2022) are by far the least likely to be users and the typical user earns a high or very high income (8000–10,000CHF or >10,001CHF). Finally, gender has no significant impact on the likelihood of being a user.

**Table 4.** Results of the binary logistic regression.

					95% CI	
	*B*	SE	Sig.	Exp(*B*)	LL	UL
Age	-.028	.006	.000	.972	.961	.984
Gender^a^	-.077	.179	.667	.962	.652	1.314
Income^b^						
Low	.175	.174	.315	1.191	.847	1.677
Medium	.083	.181	.649	1.086	.761	1.550
High	.468	.175	.007	1.597	1.133	2.250
Very high	.423	.168	.012	1.526	1.097	2.122
Education^c^						
Middle	.006	.201	.975	1.006	.678	1.493
High	-.103	.262	.694	.902	.539	1.509
Very high	-.409	.199	.040	.665	.450	.981

*B* = logistic coefficient; SE = standard error of estimate; Exp(*B*) = exponentiated coefficient; CI = 95% confidence interval for Exp(*B*); LL = lower limit; UP = upper limit.

^a^ 0  =  male.

^b^ 0  =  very low.

^c^ 0  =  low.

These results highlight the significant impact of age, income, and education on the adoption of myStep. This suggests the presence of inequal patterns that could reproduce or strengthen established social inequalities. However, the interview data (see below) and the low values of the model coefficients (*R*^2^  =  *.*069 (Nagelerke). Model *X*^2^ (8)  =  17.793, *p*  =  .023) suggest that adoption of self-tracking is tied to more complex processes and point to the limits of raw sociodemographic variables. Further in the document, we will come back to the topic of adoption and use qualitative data to refine our understanding of its logics. But first, we turn to four prototypical types of appropriation, or use, of the technology.

### The patterned use of ST technologies, analyses of interview data

We now turn to the appropriation of myStep in everyday life. How do people use the application? In the next sections, we describe four clustered (in the sense that there are similarities between users in a similar pattern) and characteristic (in the sense that what is found in a pattern is not found in others) types of myStep use that emerged from the analysis of interview data. Table 5 provides selected quotes for each type of use with regards to the codes described in the methodological section.

**Table 5.** Building use categories from coding sequences, selected quotes.

Code	Meritocrats (*n* = 8)	Litigants (*n* = 8)	Scrutinisers (*n* = 6)	Good-intentioned (*n* = 19)
Impact on behaviour	I do not think is was designed for people like me… I’m pretty fit (laughs)… So no, I did not change anything indeed (1.21) Well, I know I am going to fitness at the end of the day so… It’s probably to make people who are not very athletic more motivated (1.5)	So did you change your habits? No…no, not at all… It remained the same as before I would say (2.16) I think it can help people who are not yet aware, and who are maybe lazy (1.12)	Well, I’ve always forced myself to do some sport, it hasn’t changed in an extreme way (1.13) I noticed pretty quickly that I was not the target audience. But you can always learn anyway (1.14)	I try to do gardening, I walk inside the garden, and I also went go for walks, more than I usually did (1.2) I did not walk to the train before. And I realized that I’m actually faster So now I walk to the train almost every day, unless it’s raining hard. 1.3
Relation to incentives	Did the incentive motivate you ? No, no not really (1.21) I get some, well I get a few francs a month but it’s not… it’s not much, but why not? (2.3)	I started because, well, it is 40 cents a day… (2.19) For me the motivation is financial. I think it’s important that… I mean, I’m always annoyed at paying for people who do not take care of themselves (2.16)	It’s just a few cents. I do not do it for this, I do it for me. It’s like everywhere. The chances of achieving a goal without recognition are higher (1.8) I think it is 40 cents for 10,000 steps, so it is in any case not financially that I signed up for this application (2.5)	The incentive, well it may sound silly, but it was not important for me (1.22) (laughs) I broke up two years ago… before I wasn’t the one managing all the insurance stuff and all that. I discovered mystep. It is a financial issue too (2.10)
Relation to physical activities	I’m pretty, I’m very competitive and so having those numbers is important because it allows you to measure your progress (2.1) I’ve always done sports and I enjoy doing it. I don’t do sports because of the technology, it came after that (2.2)	I’m not one of those people who look for their limits in sports. I don’t really like intense sport. I think it’s good to integrate sport into everyday life (1.12) I have been a sportsman since I was a child, I have been playing ice hockey since I was 4 years old, I have never stopped. (2.16)	I’ve always done sports, it’s good for me. Being outside, smelling the air, even if it’s only fifteen minutes, it’s nice (1.7) I move around a lot, I go jogging, in-line, skiing, things like that… really, on a regular basis. I’m a bit older but all in all…I can’t live without sport (1.14)	So I don’t really do sports, but rather movement (Bewegung). I used to ride my bike to work, I walked a lot (1.1) Mmm… To start a real sport again would be good for me, Yes, I would like to move more, but not all at once, rather quietly (1.6)
Relation to technology	I’m interested in technological development. I already have a lot of apps. And I’m interested in knowing what exists, what’s available (2.1) I use the polar platform too, in a more precise way, as a training journal (2.2)	I kind of like technology. I used to have a watch for running and I was always looking at my curves on the computer (1.12) I’ve been using heart rate monitors for many years. At first, they were simple polars that only showed the beats. now we have mystep and the link with the insurances (2.16)	Yes it’s a bit stupid, but for me the motivation was that it is a technology. There is a bot, it’s fun, yes, try it out! (1.7) Yes, I am (interested in technologies). I’m always looking to see if there’s anything new. But the risk, when you’re retired, is to miss things. If you don’t keep up, you quickly get lost and it goes so fast, afterwards. But for the moment it’s going ok yes (2.5)	Honestly, I keep the fitbit just because they reimburse 40 cents. The day my fitbit dies I don’t think I’ll take another one (2.14) Well, there are excellent tools, but apart from that, I am not a big fan. I am not seeking the latest gadget. I benefit from them but also endure them for the most part, like everybody… I can really do without them for a week. (2.9)
Relation to numbers	I’d rather run on a track where I know it’s 400 meters and I can measure myself. For me, it’s more important to know what I’m doing than to just… do it… randomly (2.1) It’s really information I would say. Like the meter, like the instruments you have in a car in fact (2.2)	It’s an important because if you don’t use measures you’re going to run badly, you’re going to hurt yourself, and above all you’re going to train badly (2.16) It gives me some information because I also have the pulse so I can adjust activities. You have to find the right measure (2.5)	And then the stats…it hardly gives me any stats…there is almost nothing. Not even time, “when did I move the most, when did I move the least?” Yes… a little more data. (1.7) Yes, well, at the beginning it used it to correlate the distances traveled with the number of steps (2.6)	Yeah, yeah it also calculates the floors covered plus the distance. Nothing transcendent. It should be permanently connected to my phone to load the data but I tend to forget that (2.9) I’d be interested to see the whole cardio aspect. And then uh, especially cardiac coherence, I think there are some people who are starting to be very advanced on that. But it’s expensive and it would be more of a… I don’t really need it, but I find it interesting (2.4)
Solidarity/responsibility	I think that there is a current trend that people don’t do anything until they are told to do it. I’ve often had that feeling that there is this tendency in our society. (1.10) I think we need to individualize a little bit more. That could push some people to change their habits and through that change the total cost of health and benefit everybody (2.1)	Why do I have to pay more for my health insurance while the other smokes a pack of cigarettes a day. I have never smoked a cigarette in my life. Why do I ride my bike to work every day? (2.16) People who complain, who don’t move, who don’t do anything and then… who are very sedentary and who fall ill; on the one hand, I… it’s perhaps a bit hard. I’m not going to say, ‘that’s what you get’, but it’s a bit your responsibility, it’s a bit up to you… (2.7)	It’s a bit dangerous. I’m against fining people who are inactive We should not discriminate. I’m more in favor of strong solidarity in insurance (1.14) I think that the idea of modifying behaviors is excellent if it remains an incentive and not a stigmatization or a punishment (2.6)	Yes, I think it could disadvantage other people who are already prejudiced by other things. If on top of that they have to pay more for health insurance. It’s already a heavy burden (2.14) I’m reluctant because I think there may be so many cases where people who are not active cannot do anything about it. And then they would pay even more. I would be afraid that it would create more disparities (2.4)
Health	I’m interested in fitness also in connection with food. Eat healthy, drink enough, don’t eat too many carbs, lots of protein, protein from different sources. Not only red meat but also fish. Or nuts. Lots of vegetables and fruits, all of that. Fiber. Little alcohol, rarely. Yes, all of that. (1.5) Because I have, fortunately, no diseases, no infirmities, I feel that my bones and my body are…stable and strong because of physical activity (1.10)	I am into sports; I know that I don’t have any problem anyway. My physical condition is good (2.9) I think it can help people who are not yet aware, and who are maybe lazy. That, I think, can be good. But for someone like me who already moves a lot or does sports regularly, it’s probably not the right tool (1.12)	Well, I pay attention to my health, I am young, but I pay attention. For me health is important. I just find that the approach only with steps is a bit limited (1.7) I thought, well, I’ve got good health, but it’s not going to last without problems. And well, yes, moving around a lot is always good. And so, I wanted to know how many steps I was taking (1.8)	I had a few health alerts, so I said to myself ‘I have to…’ and I had gained between 10 and 15 kilos. I couldn’t go back down. At the beginning of the year I said no, now I am 50 years old, I have to get in better shape for my health. And that’s how I got started (2.12) After the hospital, I spent six weeks at home. So doing these steps every day I really felt an improvement. It really, how can I say, motivated me. And it also helped me to do more. To have something to do every day, and even on weekends, yeah (1.13)

#### The meritocrats

The first type of use is characterised by an injunction to improve. This necessity is at the core of *meritocrats*’ appropriation of the technology. It applies to other aspects of their lives, suggesting that ST is part of a bundle of practices related to a common lifestyle. When asked about the necessity to measure steps, an interviewee provides the following answer:

So that I can see where I am at! How I feel after I have done my steps, if I feel good, bad… and to go even further and run – that is the point – even further than I ever had (…) Now I walk 25, 30 (minutes) without a problem, maybe not running. But it is… yes always surpass one's, yes always go further. (Finance specialist, 45-years-old)

These users, contrary to the three other categories, are not satisfied with reaching the objective. They strive for more steps in what seems like an endless quest for optimisation. Measurements are used as a standardised baseline out of which increments, and decrements can be deduced. Hence, the necessity to measure regularly and the irritation toward walks that are not measured. The centrality of numbers and quantification is also highlighted by a disdain toward other forms of relations to physical activity, such as feelings that are described as ‘guesswork’ or ‘silly’.

If the technology is appropriated as a proxy for continuous improvement, it is also valorised as fostering ‘accountability’. From that perspective, using the application reveals the ‘real’ state of things and, thus, confronts users with their active or inactive lifestyle.

Interviewer: you mean that your wife does not like the idea of measurement? Interviewee: Ah no! But also, because numbers… they show things as they are… and it makes you accountable. If you do not see the numbers, you can always say “that is ok, I am good” (…)/with numbers/you become responsible, huh? It is written on paper, huh? You cannot escape it! (Sales manager, 51-years-old)

Accountability and responsibility are used interchangeably by *the meritocrats*. The possibility to measure is closely tied to a moral duty to face one's responsibilities to be physically active to diminish health costs. Indeed, these users consider myStep – and individual behaviour – as key to diminishing the global costs of health.

All users in this category are highly educated, earn a high income and work in private companies (mostly in finance). Moreover, they have been socialised in sports and/or professional environments that valorise competition, improvement, and quantification.

Well, it also has to do with my work. Most of the time, my job is to make profit on money, on my acquisitions. It is always about profits, gains. And it is physical, tangible, I know exactly what I win. And here I know exactly what I win too; in steps, in cardio, in minutes, I think that… for me it is related. (Finance specialist, 45-years-old)

*The meritocrats* have incorporated – prior to their appropriation of myStep – an ethos that valorises improvement and quantification. They already reach their daily step objectives before using the app and thus only slightly change their behaviours with improvements that are not rewarded (steps over 10,000 are not rewarded). The centrality of improvement, which is absent from the script of the application and from the other three forms of appropriation, seems to stem from these users’ lifestyles, refracting the dispositions they have acquired in specific environments, notably work in private finance companies and competitive sports. This may be tied – and the same could be said, *mutatis mutandis,* for the next two types of use – to what Bourdieu calls *amor fati* or necessity made virtue; that is, instances where ‘external constraints have been transformed in interior models, in personal tastes (or passions)’.^44^ Here, the script of the application dynamically interacts with the lifestyle of users and allows for the expression of incorporated dispositions.

Centration on improvement and optimisation in ST is often interpreted by scholars through the notion of entrepreneurial self^45^ which highlights individual's managerial attitude towards their bodies, health, and lives.^46^ In this perspective, ST is both the product and productive of neoliberal trends in which the burden of responsibility for health is transferred from institutions to individuals.^47^*The meritocrats* in our sample have internalised this entrepreneurial ethos in specific work and sport environments and find in myStep both a way to express and reinforce it. Let us note that similar forms of appropriation have been documented by Kristensen among Danish trackers.^48^

#### The litigants

*Litigants* never mention improvement. Moreover, they do not seek change. None of the users in this category changed their walking behaviour as part of the program, and they all reached their objective since day one.

Interviewee: … but all of that /walking/, I was doing it before. It has nothing to do with the app; I was doing it before.

(Nurse, 41-years-old)

This leads to the following question: why do they use the app? The first answer is valid for all users in this category; they seek confirmation that their behaviour is aligned with the expected lifestyle or norm that the technology encompasses.

Interviewer: You mentioned the pleasure to see the globality?

Interviewee: Yes, it is pleasurable yes…

Interviewer: Why is it important?

Interviewee: It is a confirmation … When all the boxes are green, I know that I was not lazy! (Nurse, 41-years-old)

For *litigants*, aligning with the objective is reassuring or securing. They use myStep as an actionable (daily and for a financial reward) classificatory scheme, legitimised by institutions, which ‘implies a symbolic privilege: the privilege of being comme il faut, conforming to the norm’.^49^^(p69)^ On the other hand, people who do not comply with the norm are condemned. The – morally laden – word ‘laziness’ is indeed used to refer to a potential failure to reach the objective. Daily objectives are understood as moral compasses, and *litigants* harshly criticise people who are inactive and do not comply with prescribed norms. Like *meritocrats*, *litigants* consider that movement is a matter of individual responsibility. They perceive myStep as a fairer way to determine insurance premiums.

For me it's something that's important and so… people who complain, who don't move, who don't do anything and then… who are very sedentary and who fall ill; on the one hand, I… it's perhaps a bit hard. I'm not going to say, ‘that's what you get’, but it's a bit your responsibility, it's a bit up to you… (School teacher, 31-years-old)

The insistence on morality and justice, coupled with an emphasis on rewards and objectives, is characteristic of *litigants*’ appropriation of myStep. It explains why they are the only category that mentions potential forms of cheating:

People may give their phone to someone else. I never did it, but, well… It is always a danger when one gives people money; they will try to circumvent things. And there, I think we need to be strict: it is fraud. (Banker, 70-years-old)

In contrast to the first category, it comprises many people with unstable or difficult financial conditions. Users here fall into two categories: people with favourable life conditions who adhere to neoliberal political values (but have not incorporated a need for improvement or optimisation) and people who struggle with difficult life trajectories (migration, unsatisfactory financial conditions), share the individualising logic of myStep and need the financial reward.

The appropriation of myStep is centred on the objective, which is understood as a norm or a moral compass. Users in this category adopt the application to reassure themselves that they comply with a norm and turn its logic against ‘lazy’ individuals who do not. myStep becomes a means to contrast one's identity with others. Numbers and the application – which are legitimised by their integration into an insurance company program – provide a quantitative, practical, and tangible tool to denounce abnormal behaviours, drawing boundaries between people and expressing one's morals on a daily basis.

#### The scrutinisers

Interviewer: Let us talk about myStep. I see that you have brought… (designates papers that the interviewee has laid on the table).

Interviewee: Numbers!

Interviewer: Numbers… well let us start from that then!

Interviewee: So, those are the statistics that I have produced since 2016. I pulled the results by month for each year. And we are there (points to the current month on the curve). And you remember when I talked about joint pain? We can see, here, that there is a trend towards decrease… (Expert in information security, 64-years-old)

*Scrutinisers* have an analytical relationship with numbers, which are seen as partial evidence for their quest to describe and analyse their behaviours. These users aggregate numbers in curves (myStep already does it, but not on the same scale) or correlate them with other numbers (weather forecasts, speeds, heart frequencies, etc.). They are sceptics, but not of numbers; they are passionate about numbers and never criticise the application's overall quantification rationale. They are sceptical about each tool and each metric. It is as if they are looking for the best combination of metrics to account for their activities.

Interviewer: Do you sometimes go for a walk without the watch?

Interviewee: It happened, but it became a real impulse, to think ‘ah where am I at? 8000? 2000?’ Ah, it is growing, it is… and I am always thinking about a potential correlation, meaning, on another path. How much time does it represent? That is easy, I am used to it. And the number of steps…? From which I deduce distance, because when you know the average stride length… (Expert in information security, 64-years-old)

This specific point of view is different from the first category in two main ways. First, numbers are, as we have seen, transformed, pooled, and distrusted at first sight. Second, the overall goal is not to improve but to understand or to monitor. There is an intrinsic pleasure in measuring and trying to figure things out.

This analytical relationship with numbers and myStep stems from a specific professional socialisation. All users in this category have been trained either in the fields of science or engineering. Once again, the appropriation seems to be framed by user's specific education. Let us note that users in this category share similarities with members of the Quantified-Self movement, an institutionalised group of passionate users who also tend to originate from the fields of engineering and science.^18^

Interviewee: So, mainly heart rate and speed… That's basically it, but it's true that it's also linked to my work, because we only do measurements, tests, statistics and… you see how. We know that if there's a breakdown, we have to analyse several things; we want to say yes, that's it… That's a bit of it; it's the side that's maybe scientific or yeah… (Laboratory technician, 70-years-old)

For the *meritocrats*, *litigants*, and *scrutinisers*, use of the technology leads to the reproduction of already existing tastes more than to modifications of behaviour. The constant necessity to improve and optimize,^48^ the biomedicalization of everyday life and the quantification of health and bodies,^50,51^ were acquired in prior socialisations for the *meritocrats* and the *scrutinisers*, and are re-enacted and strengthened by the intervention. In the *litigants*’ case, the intervention reinforces an already present normative view of health and facilitates an incorporated disposition to draw boundaries between responsible and irresponsible citizens.

### Life trajectories as enablers for change

#### The good-intentioned

The clearest characteristic of people in this category is that their interest in technology, measurement, sports – and, more generally, in myStep itself – is low. This is particularly striking; notably, in comparison to the other three other patterns, and points to a misalignment between the script of the application and these users’ dispositions.

Well, there are excellent tools, but apart from that, I am not a big fan. I am not seeking the latest gadget. I benefit from them but also endure them for the most part, like everybody… I can really do without them for a week. (School teacher, 37-years-old)

Contrary to the three other categories, here, adherence to quantification and technology is low, with people often describing the process as tedious. The other common thing among the *good-intentioned* is that they all have directly or indirectly encountered health-related problems. In the following quote, the interviewee simply refers to feeling less healthy. But other cases include living with someone who has a serious heart condition, having undergone surgery, being pressured by close – and worried – relatives, etc.

The reason is that since I am retired, I do not go to work with my bike every day. It felt like that daily movement was lacking. I became less healthy and put on weight. So, I decided if I do not bike every day anymore, I have to find a way to move. (Insurance manager, 64-years-old)

*Good-intentioned* focus on changing their behaviour. They see myStep as a support to bring some physical activity in their daily lives. This is unsurprising considering that their appropriation has to do with concrete health issues. They truly strive to walk more; something that cannot be said of users in the three preceding categories.

Yes, ten thousand steps is a lot. But, as it is planned, you have to do it. You can do it under pressure (laughs) or… Sometimes it is good because you do a little more. Sometimes it is bad because you are always focused on it. (Medical secretary, 57-years-old)

The hesitation in this user's experience is notable. She describes a form of pressure that is sometimes good for it helps her reach her objective. However, sometimes it is bad; it draws her focus and attention from other things. Here, we find a struggle that is characteristic of attempts at self-discipline. Numbers and the technology serve as a support or a control/pressure to perform a desired behaviour.

Yeah, well, sometimes you tell yourself: okay, let's walk there. Yeah maybe… You feel like you want to do a few steps today. Or, sometimes, when you start parking, you tell yourself, because you always try to park as close as possible… And, sometimes, now it is reversed. You tell yourself, okay I will go for a small walk; it feels good, and I get fresh air. So, you park a little farther away once you find a place… And then… sometimes, you think slightly differently about “car-parking” I think, maybe… (Team manager, 49-years-old)

This category is characterised by mixed socioeconomic status and heterogeneous socialisations. The common denominator is a life trajectory where health problems play a role. It seems that encounters with serious health issues flatten the differences that may be observed otherwise. Thus, dispositions here seem to be – at least partly – overwritten by a life trajectory that entails health difficulties. These users strive to adopt a practice that is not aligned with their dispositions; hence, the sense of going against oneself, of struggling to force oneself.^44^ A connection can be made with Bourdieu's description of goodwill to refer to petite bourgeoisie's tendency to ‘force’ their tastes and impose themselves what is considered culturally legitimate.^33^

For the good-intentioned, ST supports forms of self-disciplining which can lead to the promised change in behaviour. The interaction between myStep and users whose life-trajectory entails health issues – or worry that health issues may emerge – leads to actualisations of the technology's behaviour change components. In those cases, our results are in line with ST studies highlighting the tensions and efforts that users face, and the work they must perform, when striving to change themselves.^19,52^ For good-intentioned users, the promise of ST to change behaviour is implemented via an attempt at self-discipline that could be analysed – but this would go beyond the reach of our article – through a post-Foucauldian perspective.

Nevertheless, some cautions are in order. First, according to the insurance, only 1% to 2% of the customers use the application, so the overall impact on population behaviour is likely to be weak compared to what is promised. Second, interviews suggest that good-intentioned users struggle with the attempt to change behaviour and may not succeed in implementing it. Data is currently lacking regarding the long-term consequences of ST on behaviour modification.^28^

### Interviews and adoption, the role of social dispositions and trajectories

In this final result section, we come back to the topic of adoption, using qualitative findings to complement the results of the binary logistic regression. We have learned that for *the meritocrats*, adoption of the technology is driven by an ethos that emphasises self-responsibility and improvement. In other terms, users adopt *myStep* because it aligns with an existing lifestyle oriented towards self-optimisation. A similar process leads *the scrutinisers* to adopt the technology. In their case, adoption of the technology inscribes itself in a disposition to measure things and analyse data. Let us note that for these two first categories, and contrary to the other two, adoption is not tied to the financial rewards distributed by the application, which are regarded as unimportant. This is likely tied to the social position of these users, who all earn high incomes.

This is not the case of the next two categories, where users’ economic profiles are much more heterogenous. *The litigants* adopt the technology because they believe in its political underpinnings, they regard *myStep*'s risk categorisation as fairer for it financially acknowledges their efforts regarding physical activity. Let us note here that for these first three categories, changing behaviour is not the main issue. Most of these users already reach the step objectives prior to the adoption of *myStep* and already consider their lifestyles healthy. To the contrary, changing one's walking behaviour is central to the adoption of the technology by the last category, *the good-intentioned*. They come from heterogenous backgrounds but face similar social conditions. For them, the appropriation of *myStep* takes part in the reconfigurations of individuals lives associated with chronic illness trajectories.^53^

These results suggest that adoption is explained by the interaction between social determinants, secondary socialisations, and life trajectories.^35^ That perspective explains the difficulty faced by current quantitative studies (including ours), which limit their models to broad social indicators. Consideration of these proves the presence of a statistical divide in adoption but fails to account for the various logics that underly it. Our use of both quantitative and qualitative data hopefully paves the way for quantitative studies that takes the social grounds of adoption into account.

## Discussion

### Main findings: patterns of adoption and use

Our results reveal homologies between both adoption and use of the ST technology on the one side, and users’ social backgrounds on the other.^54^ They demonstrate that poorer, older, and less educated people are less likely to adopt the technology. Moreover, we distinguished four categories of users who engage with the technology for the following reasons: tendencies to gravitate towards optimisation and competition, social norms, quantification, and the threat of health conditions. In the three first cases, professional socialisations and life trajectories tend to determine the mode of appropriation, whereas, in the fourth category, a disruption of the life trajectory seems to be the main trigger.

Another important finding is that the goal to change walking behaviour is only central to one category. That category, the *good-intentioned*, brings together people with heterogenous socioeconomical backgrounds who often suffer from health conditions. As a result, they attempt to establish a new organisation, or management, of the disease.^53^ This pushes them to go ‘against themselves’ (they were not particularly disposed to use a technology or be active before they developed a health condition) to adopt healthier lifestyles and thus mitigates the influence of their past dispositions. The fact that disease – a major disruption in life trajectory – seems to open a window of opportunity for behaviour change is an important finding for the design of future ST interventions. In the case of the three other categories, the tracking technology offers a possibility to express and enact an already incorporated ethos. In other terms, the self-tracking technology is incorporated to a broader set of practices that respectively emphasize optimisation, normative behaviours, or quantification.

Our results confirm recent findings that emphasize the role of structural factors in ST adoption.^29,30^ In addition, a few studies have highlighted different styles of tracking but remained impervious to the social origins of these styles, notably because they mixed different technologies used in different contexts.^25,55,56^ Our results also substantiate the fact that those among the poorer and less educated who still opt for the technology are partly driven by the financial rewards, which bears the risk of making opting out of such technologies a social privilege,^9^ thereby mitigating the promises that surround ST technologies.^3^ Regarding the first three categories, users do not actively seek to change their walking behaviour. Here, the use of ST technologies tends to echo users’ tastes^54^ and contributes to their reproduction (or reconfiguration) and does not, as promised by ST proponents, contribute to changing lifestyles. However, we cannot conclude that the intervention is useless with regards to public health objectives, for the use of the technology to express and enhance a specific ethos may drive the maintenance of walking behaviour.

Patterned heterogeneity in the diffusion of ST raises questions about the intervention's actual health promotion potential. In other words, the promises of ST programs to change people behaviour and better their health seem to be undermined by classical socio-determinants of health.^57^ As in many other cases, the people who are more likely to use the application are not the one typically identified as the main targets of health promotion.^58^ Let us note that we should remain cautious regarding that interpretation for the category of non-user does not automatically equate inequality or disadvantage.^59^ Current health policies, notably fuelled by neoliberal individualism and speculative promises regarding technologies,^2^ tend to lean on digital behavioural interventions, despite strong evidence that their utility is limited when addressing the impact of social inequalities.^60,61^ ST interventions require precise and informed targeting if they are to foster the objectives of health promotion.

### Contribution to the critical literature: the role of social backgrounds

Our results suggest that ST should be considered as a cultural practice that entangles itself with already existing practices and lifestyles. We argue that technological tools and practices should not be analysed as autonomous categories. Changing people's behaviours is one of the main promises of digital tracking interventions, which eventually entails changing people's dispositions (here towards physical activity) and practices. Our results, therefore, turn the promise of digital tracking on its axis; we have shown that ST is often transformed by and appropriated according to people's dispositions. Consequently, users’ dispositions should be systematically considered in the development, study, and evaluation of ST programs. This is not to say that technology, peers, context, and environment do not also play a role in appropriation, but that social backgrounds are most often neglected. Something that is generally induced by the dominance of post-Foucauldianism and STS in critical studies, or behavioural psychology in human-computer interaction studies.

Our research adds to two currents of the literature on self-tracking. First, it contributes to the nascent quantitative literature that aims to identify the profile of self-trackers. So far, this literature has mostly been descriptive.^29,30^ Our research furthers the discussion by proposing a systematised theoretical framework. Our use of Bourdieu's framework highlighted the role of dispositions in the adoption and use of ST technologies, adding to the sole consideration of broad sociodemographic variables. Moreover, focusing on a single application deployed in an institutional context allowed us to go beyond the description of idiosyncratic uses and reflect on ST's entanglement with inequalities. Second, our research adds to the more established literature on self-tracking. This literature is characterised by the dominance of Foucauldian studies, STS and post-phenomenology. These frameworks led to elaborate accounts of ST but tend to neglect the role of users’ social backgrounds. Our research brought the focus back on these, paving the way for more symmetrical analyses.

## Limitations

Focusing on dispositions with a Bourdieusian lens has some limitations. Meanings and normative models of collective identity can be influenced by several forms of socialisation. Moreover, technology itself plays a major role in the process. In this article – because all users used the same application – we slightly obscured the role of technology to focus on users. But we do not neglect the fact that all the described patterns are entanglements of users and technologies.^62^ Also, giving attention to dispositions and lifestyles should not lead to depicting users as passive.^63^ Lastly, the emphasis that Bourdieu put on reproduction and structures may – falsely – lead to essentialist and overly deterministic accounts, which we hope to have avoided.

In this article, we developed categories regarding the use of technology. These are based on empirical data and meant to highlight the variety of appropriations. However, such constructs come with the following limitations. a) The proportion of people in the categories cannot be taken for representative of the whole population, b) some individuals are close to the ideal types, but others are more on the periphery of categories or can be situated between two categories, and c) users and non-users can switch categories (notably depending on their health, especially within chronic illness trajectories). Regarding methodology, we would like to point out the following limitations. First, we did not have access to the step-count of the interviewees. Data regarding behaviour is derived from the interviews. Second, the response rate for the questionnaire was low, which may indicate that certain populations (notably unmotivated by ST) may not be represented. Finally, people without a supplementary insurance could not participate to the study. Knowing that this is generally a disadvantaged population,^64^ we may hypothesize that the inequal diffusion of myStep is even more pronounced than our results suggest.

## Conclusion

As institutions promote the promises of ST, it is crucial that scholars do not feed these promises by forgetting to reinscribe ST into its social grounds, which are both diverse and unequal. Otherwise, the risk is to transform socially grounded forms of reproduction and exclusion into individual lack of will or technological failure. Overemphasising ST's transformative potential may lead to relegating social determinism to the background and transmuting differential dispositions into personal (lack of) responsibility for health. In that sense, both scholars and institutions may contribute to reinforcing forms of symbolic violence towards those who do not adopt those new technologies and thus feed broader neoliberal regimes.

## Notes

a. In the fields of health and physical activity, these terms generally refer to the measurement of bodily functions via connected tools.

b. Scholars have shown that digital intervention in insurance programs may—for now—be attempts at improving customer loyalty more than real attempts to change behaviours or identify risks.^5,65^ Nevertheless, in this article, we decided to tackle the promises regarding health promotion—which are currently pervasive—and evaluate them empirically.

c. In Switzerland, basic health insurance is mandatory and covers a range of strictly regulated rights. Supplementary insurance—as its name suggests—refers to insurance contracts that cover additional and nonmandatory health insurance options. Both are offered by private companies.

d. Users obtain a 0.20 CHF/0.40 CHF deduction on their supplementary insurance premiums each day when they reach the objectives of 7,500/10,000 steps.
